# Predicting the international spread of Middle East respiratory syndrome (MERS)

**DOI:** 10.1186/s12879-016-1675-z

**Published:** 2016-07-22

**Authors:** Kyeongah Nah, Shiori Otsuki, Gerardo Chowell, Hiroshi Nishiura

**Affiliations:** Bolyai Institute, University of Szeged, Aradi vértanúk tere 1, Szeged, H-6720 Hungary; Graduate School of Medicine, The University of Tokyo, 7-3-1 Hongo, Bunkyo-ku, Tokyo, 1130033 Japan; CREST, Japan Science and Technology Agency, Honcho 4-1-8, Kawaguchi, Saitama 332-0012 Japan; School of Public Health, Georgia State University, Atlanta, Georgia USA; Division of International Epidemiology and Population Studies, Fogarty International Center, National Institutes of Health, Bethesda, Maryland USA; Graduate School of Medicine, Hokkaido University, Kita 15 Jo Nishi 7 Chome, Kita-ku, Sapporo-shi, Hokkaido 060-8638 Japan

**Keywords:** Middle East respiratory syndrome, Airline transportation network, Infectious disease, Risk assessment, Importation

## Abstract

**Background:**

The Middle East respiratory syndrome (MERS) associated coronavirus has been imported via travelers into multiple countries around the world. In order to support risk assessment practice, the present study aimed to devise a novel statistical model to quantify the country-level risk of experiencing an importation of MERS case.

**Methods:**

We analyzed the arrival time of each reported MERS importation around the world, i.e., the date on which imported cases entered a specific country, which was modeled as a dependent variable in our analysis. We also used openly accessible data including the airline transportation network to parameterize a hazard-based risk prediction model. The hazard was assumed to follow an inverse function of the effective distance (i.e., the minimum effective length of a path from origin to destination), which was calculated from the airline transportation data, from Saudi Arabia to each country. Both country-specific religion and the incidence data of MERS in Saudi Arabia were used to improve our model prediction.

**Results:**

Our estimates of the risk of MERS importation appeared to be right skewed, which facilitated the visual identification of countries at highest risk of MERS importations in the right tail of the distribution. The simplest model that relied solely on the effective distance yielded the best predictive performance (Area under the curve (AUC) = 0.943) with 100 % sensitivity and 79.6 % specificity. Out of the 30 countries estimated to be at highest risk of MERS case importation, 17 countries (56.7 %) have already reported at least one importation of MERS. Although model fit measured by Akaike Information Criterion (AIC) was improved by including country-specific religion (i.e. Muslim majority country), the predictive performance as measured by AUC was not improved after accounting for this covariate.

**Conclusions:**

Our relatively simple statistical model based on the effective distance derived from the airline transportation network data was found to help predicting the risk of importing MERS at the country level. The successful application of the effective distance model to predict MERS importations, particularly when computationally intensive large-scale transmission models may not be immediately applicable could have been benefited from the particularly low transmissibility of the MERS coronavirus.

## Background

Middle East respiratory syndrome (MERS) is a viral infectious disease caused by the MERS associated coronavirus (MERS-CoV), which was first identified in September 2012. Infections with this virus have been reported from countries in the Middle East where dromedary camels have been found to be an important reservoir of the coronavirus. The case fatality ratio of MERS among confirmed cases has been estimated at about 40 % [1]. Human-to-human transmission has been facilitated in healthcare settings [2] with the contribution of hospital-based transmission of MERS estimated at about 80 % using an epidemic model [3]. The pandemic risk that MERS poses is limited by the fact that *R*_0_ (the basic reproduction number, i.e., the average number of secondary cases produced by a single primary case) is estimated at below 1 and that MERS outbreaks have been greatly confined to the healthcare environment until now [4, 5]. Antibodies against MERS-CoV have been found among both dromedary camel populations and camel-exposed humans in Saudi Arabia and other countries of the Middle East [6, 7], which have often been the source of multiple MERS case importations around the world. As highlighted by the recent MERS outbreak in the Republic of Korea since May 2015 [8, 9] together with one case importation with no secondary cases into Thailand in June 2015 [10], the risk for global spread of MERS is sufficiently serious to warrant the need to find ways to assess the risk of importing MERS case around the world, because those single importations could potentially escalate into regional outbreaks that could, in turn, lead to a serious damage to economic and public health activities [11].

To support risk assessment practice on potential MERS outbreaks, it could be helpful to pre-emptively characterize the risk of importing MERS case across countries. By estimating the risk of MERS importations, one can comparatively understand the country-specific risk, and such understanding may help raise situation awareness among the general public, facilitating preventive action planning. In this context, a metapopulation epidemic model that uses the airline transportation network has been employed for predicting the global spread of various emerging infectious diseases, including severe acute respiratory syndrome (SARS), the influenza pandemic H1N1-2009 and Ebola virus disease [12–14]. These transmission models have not only helped study epidemic scenarios but also estimated the effectiveness of specific countermeasures targeting travelers, e.g., entry and exit screenings and travel restrictions [15, 16]. While the metapopulation epidemic model is simple in its structure, epidemic forecasting by fitting the model to empirical data entails optimization procedures that are often computationally intensive. Moreover, while a few published studies have already investigated the risk of MERS associated with mass gatherings in Saudi Arabia [17, 18], the distribution of secondary cases per single primary case is highly over-dispersed [4, 11] with the overall *R*_0_ falling below 1. Hence, the metapopulation type models that have been used to evaluate epidemics of communicable diseases (e.g., *R*_0_ > 1) may not be directly and immediately applied to model the global spread of MERS.

While metapopulation models are useful for capturing various aspects of global transmission dynamics, a simple yet tractable prediction model to carry out fast risk assessment by public health authorities is needed. Such a tool might just quantify the basic information, e.g. (i) the percentage of importing MERS case at a point in time and (ii) when the importation of MERS case is expected. The present study aims to devise and apply a novel statistical model to predict the risk of MERS case importations by country. We assess the predictive performance of our approach and use it to identify countries at high risk of MERS importations.

## Methods

### Empirical datasets

In order to quantify our statistical model, we used data on imported laboratory-confirmed cases of MERS as of 26 June 2015 (the latest date on which our data analysis was conducted), especially focusing on the date on which the first diagnosed case arrived in each importing country. The date of entry of imported MERS cases is hereafter referred to as the arrival time and the corresponding information was retrieved from secondary data sources including the European Centre for Disease Prevention and Control (ECDC) [19] and the World Health Organization [20]. Since it was sometimes difficult to determine if a case was the result of an importation event instead of local transmission (e.g., spillover event or exposure to an undiagnosed case), original case reports were also tracked, especially among diagnosed cases in Middle East countries [21–23]. Qatar and the Kingdom of Saudi Arabia were excluded from countries at risk of importation, because indigenous cases with the history of exposure to dromedary camels have been recurrently reported after the identification of the first case in 2012.

In addition to the arrival time of MERS case importations; three pieces of further information were retrieved. First, weekly incident counts of MERS in Saudi Arabia [24] were used to mirror the force of infection among travelers. Second, the number of flight routes between pairs of countries was obtained from the airline transportation network data. The total number of flight routes between each pair of countries has an approximate dimension of 3 times 4,600 (or with 230 nodes and 4,600 edges) and was obtained from the Global Flights Network [25] derived from the OpenFlights database as on 10 November 2014 [26]. Third, dichotomous data to identify the major religion of each country which is in common with Saudi Arabia and Qatar was obtained from the literature [27]: a country in which more than 30 % of the population is Muslim was defined as a Muslim majority country.

### Building risk models

Here we describe the proposed model aimed to predict the risk of importation in each country. Let *F*_*j*,*t*_ be the cumulative distribution function representing the probability that MERS has already been imported to country *j* by discrete day *t*. The day *t* = 0 corresponds to the date of the illness onset of first identified MERS case, and throughout the present study, we set 3 September 2015 as day zero (i.e. the date on which the initially identified case experiences symptoms) [28]. Although a few earlier cases were confirmed as MERS by inspecting laboratory data a number of days after their dates of death, dates of illness onset among deceased cases were unavailable and had to be discarded when calculating the arrival time. Using *F*_*j,t*_, the probability that a country *j* has not yet imported MERS by day *t* is

1 $$ {S}_{j,t}=1-{F}_{j,t}. $$

The daily risk of importing MERS in country *j* on day *t* is defined as

2 $$ {\lambda}_{j,t}=\frac{F_{j,t+1}-{F}_{j,t}}{S_{j,t}}. $$

Thus, we have

3 $$ {S}_{j,t}={S}_{j,0}{\displaystyle {\prod}_{k=0}^{k=t-1}\left(1-{\lambda}_{j,k}\right)}. $$

We parameterized the daily risk *λ* by examining the statistical performance of different types of model parameterizations. In all models that we examined, we use the so-called “effective distance”, initially proposed by Brockmann and Helbing [29]. The metric is derived from the airline transportation network, originally based on itinerary data, by using the transition matrix and length of paths between countries. The effective length of a path {*n*_1_, *n*_1_, ⋯, *n*_*L*_} is given by

4 $$ L- \log {\displaystyle {\prod}_{k=1}^{L-1}{P_{n_{k+1}}}_{n_k}}, $$

where *P*_*ji*_ denotes the conditional probability that an individual that left *i* moves to *j*. (Note that ∑_*j*_*P*_*ji*_ = 1). Assuming that the number of passengers is identical among all international flights, the transition matrix is calculated as ${P}_{ji}=\frac{m_{ji}}{{\displaystyle {\sum}_k}{m}_{ki}}$, where *m*_*ki*_ is the number of direct flights from *i* to *k* per unit time derived from open source data [25]. Finally, the effective distance *m*_*j*_ of a country *j* from Saudi Arabia is calculated as the minimum of the effective lengths of all paths that go from Saudi Arabia to the country *j*. The effective distance, as calculated from the abovementioned process, has been known to exhibit strong linear correlation with the arrival time of SARS and H1N1-2009 across the world [29].

Assuming that the effective distance is a critical indicator of the risk of disease spread, the simplest model 1 that we examined was parameterized as

5 $$ {\lambda}_{j,t}=\frac{k}{m_j}, $$

where *k* is a constant. Namely, the hazard is an inverse function of the effective distance. As an alternative model, the information of Muslim majority countries is added, labeling corresponding countries at greater risk as compared with other countries, because countries sharing the religion with Saudi Arabia may be at greater risk of exposure to cases (e.g. through Hajj). As a consequence, a linear weight *α* is given on Muslim majority countries, i.e.,

6 $$ {\lambda}_{j,t}=\left\{\begin{array}{c}\hfill \frac{\upalpha k}{m_j}\kern1.5em \mathrm{if}\ j\ \mathrm{is}\ \mathrm{Muslim}\ \mathrm{country},\hfill \\ {}\hfill \frac{k}{m_j}\kern1.5em \mathrm{otherwise}.\kern5.25em \hfill \end{array}\right. $$

In models 3 and 4, we additionally use the incidence data of MERS in Saudi Arabia over time. It is natural to assume that the daily risk of importation is proportional to the force of infection in Saudi Arabia, and thus, the incidence of MERS, i.e.,

7 $$ {\lambda}_{j,t}=\frac{k}{m_j}{I}_t, $$

as the model 3, where *I*_*t*_ is the incidence of MERS in Saudi Arabia on day *t*. Since the original incidence data were recorded weekly (while our model is written on the daily basis), the weekly incidence was transformed to the daily data assuming uniform distribution of incidences within each week. Model 4 incorporates both of abovementioned factors into the model, i.e.,

8 $$ {\lambda}_{j,t}=\left\{\begin{array}{c}\hfill \frac{\upalpha k{I}_t}{m_j}\kern1.5em \mathrm{if}\ j\ \mathrm{is}\ \mathrm{Muslim}\ \mathrm{country},\hfill \\ {}\hfill \frac{k{I}_t}{m_j}\kern1.5em \mathrm{otherwise}.\kern5.25em \hfill \end{array}\right. $$

### Statistical estimation and assessment

To estimate model parameters, a maximum likelihood method was employed. For the countries which have already imported MERS by 26 June 2015, we used the arrival time *t*_j_ to fit the probability mass function of time at which the first importation event occurs, given by the product of ${\lambda}_{j,{t}_j}$ and ${S}_{j,{t}_j}$. Countries that have not imported MERS cases were dealt with as the censored observation. The total likelihood was

$$ L\left(\boldsymbol{\uptheta}; {\mathbf{t}}_a\right)={\displaystyle {\prod}_{j\in I}{\lambda}_{j,{t}_j}{S}_{j,{t}_j}{\displaystyle {\prod}_{j\in U}{S}_{j,{t}_m},}} $$

where *I* is the set of index of countries which imported MERS at arrival time *t*_j_ and *U* is the set of index of countries which are MERS-free by the date of analysis of 26 June 2015 (*t*_m_ = 1039 days after MERS onset). Assuming that the dichotomous information of Muslim majority country was always available, the penalized likelihood is comparable between models 1 and 2 and also between models 3 and 4. We calculate the Akaike Information Criterion (AIC) for these comparisons [30].

Once the risk model was quantified, we assessed the diagnostic performance of our model in predicting the risk of importation by employing the receiver-operating characteristic (ROC) curve and measuring the Area under the curve (AUC) [31]. For each model, the optimal cut-off value of estimated risk was calculated in predicting the importation as on *t*_m_ =1039 days using the Youden index, and sensitivity and specificity were estimated. In addition, a prediction of the risk of importation across countries for 3 years since the emergence (*t* = 1095) was computed for illustration.

### Ethical considerations

The present study reanalyzed the publicly available data from ECDC and WHO the latter of which collected the notification data from member state countries which have obtained ethical approval and written consent from patients adhering to the International Health Regulations. The secondary data were de-identified by these organizations in advance of our access. As such, the datasets that we handled do not involve any patients’ data and the present study has been exempted from the ethical approval.

## Results

**Fig. 1:**
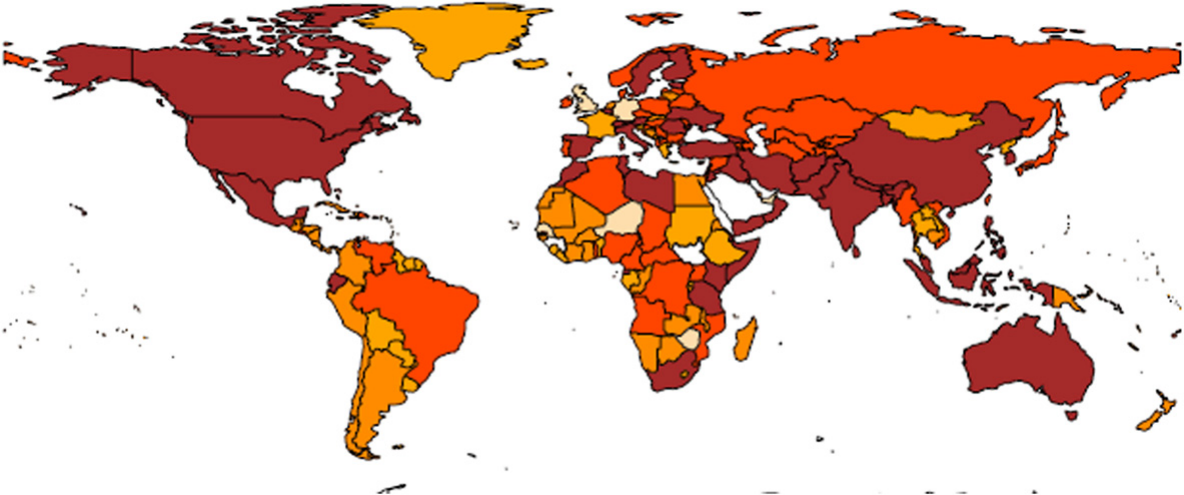
Global distribution of the effective distance from Saudi Arabia. Effective distance from Saudi Arabia. Quartiles of effective distance are differentiated by color density. *Dark brown* represents countries with the short distance from Saudi Arabia. *Orange* represents the second shortest quartile, followed by *brownish yellow* and *light brownish yellow*. The map was drawn by the authors using statistical language R (https://cran.r-project.org/)

Table 1 lists the countries and their corresponding timing of MERS case importations by 26 June 2015. In total, 24 countries have experienced at least one MERS laboratory-confirmed case importation, with the arrival time ranging from 8 to 1015 days, with a mean of 537 days and standard deviation of 271 days. Figure 1 shows the global distribution of the effective distance from Saudi Arabia. Middle East countries, United States and South Korea appeared to belong to a quartile with the shortest distance from Saudi Arabia.

**Table 1:** Timing of documented case importations of Middle East respiratory syndrome (MERS) around the world

Country	Date of arrival	Days since 3 September 2012
United Kingdom	2012/9/11	8
Germany	2012/10/24	51
United Arab Emirates^a^	2013/3/8	186
France	2013/4/17	226
Tunisia	2013/5/3	242
Italy	2013/5/25	264
Oman^a^	2013/10/26	418
Kuwait^b^	2013/11/7	430
Yemen^a^	2014/3/17	560
Malaysia	2014/4/7	581
Philippines	2014/4/15	589
Greece	2014/4/17	591
Jordan	2014/4/19	593
Lebanon^a^	2014/4/22	596
United States	2014/4/24	598
Egypt	2014/4/25	599
Iran^a^	2014/5/1	605
Netherlands	2014/5/10	614
Algeria	2014/5/28	632
Austria	2014/9/22	749
Turkey	2014/10/6	763
South Korea	2015/5/4	973
China	2015/5/26	995
Thailand	2015/6/15	1015

The date at which an infected individual has initially entered is shown

^a^For these Middle East countries, illness onset date of first identified case was used as the arrival date; ^b^There were two cases in the first instance among which the second identified case had a history of travel to the Kingdom of Saudi Arabia. The second case was thus defined as the imported case

Based on the arrival time information, all four models were quantified (Table 2). The models 1 and 3 yielded the best predictive power as measured by the AUC. In model 2, the hazard of Muslim majority countries was estimated to be 2.5 times (95 % confidence interval (CI): 1.1, 5.7) greater than that of other countries. Model fit was improved by including the information of Muslim majority countries (because of lower AIC compared to the similar model without including religion information), but AUC values of models with religion were smaller than models that did not incorporate religious majority data. Moreover, inclusion of the MERS incidence data in Saudi Arabia did not improve AUC values compared with models that do not incorporate the incidence data. Sensitivity of all models was 100 % (95 % CI: 88.3, 100.0), while specificity was 79.6 % (95 % CI: 74.0, 85.2) for models without religion and 69.2 % (95 % CI: 62.8, 75.5) for models with religion.

**Table 2:** Goodness-of-fit and diagnostic performance of risk models for predicting importation of the Middle East respiratory syndrome (MERS)

ID	Model	Number of parameters	AIC^1^	AUC^2^	Sensitivity (%)	Specificity (%)
1	Effective distance only	1	464.1	0.95 (0.54,1.00)	100.0 (88.3, 100.0)	79.6 (74.0, 85.2)
2	Effective distance + religion	2	461.2	0.87 (0.46, 1.00)	100.0 (88.3, 100.0)	69.2 (62.8, 75.5)
3	Effective distance + incidence	1	357.2	0.95 (0.54, 1.00)	100.0 (88.3, 100.0)	79.6 (74.0, 85.2)
4	All pieces of information	2	354.7	0.87 (0.46, 1.00)	100.0 (88.3, 100.0)	69.2 (62.8, 75.5)

95 % confidence intervals (CI) are given in parenthesis. 1. AIC, Akaike information criterion [30]. Note that the data used for parameterizing models 1 and 2 were different from those used for models 3 and 4, and thus, the comparison can be made only between models 1 and 2 and between models 3 and 4, respectively; 2. AUC, area under the curve, derived from the receiver operator characteristic (ROC) curve [31] to predict the risk of importing a MERS case

**Fig. 2:**
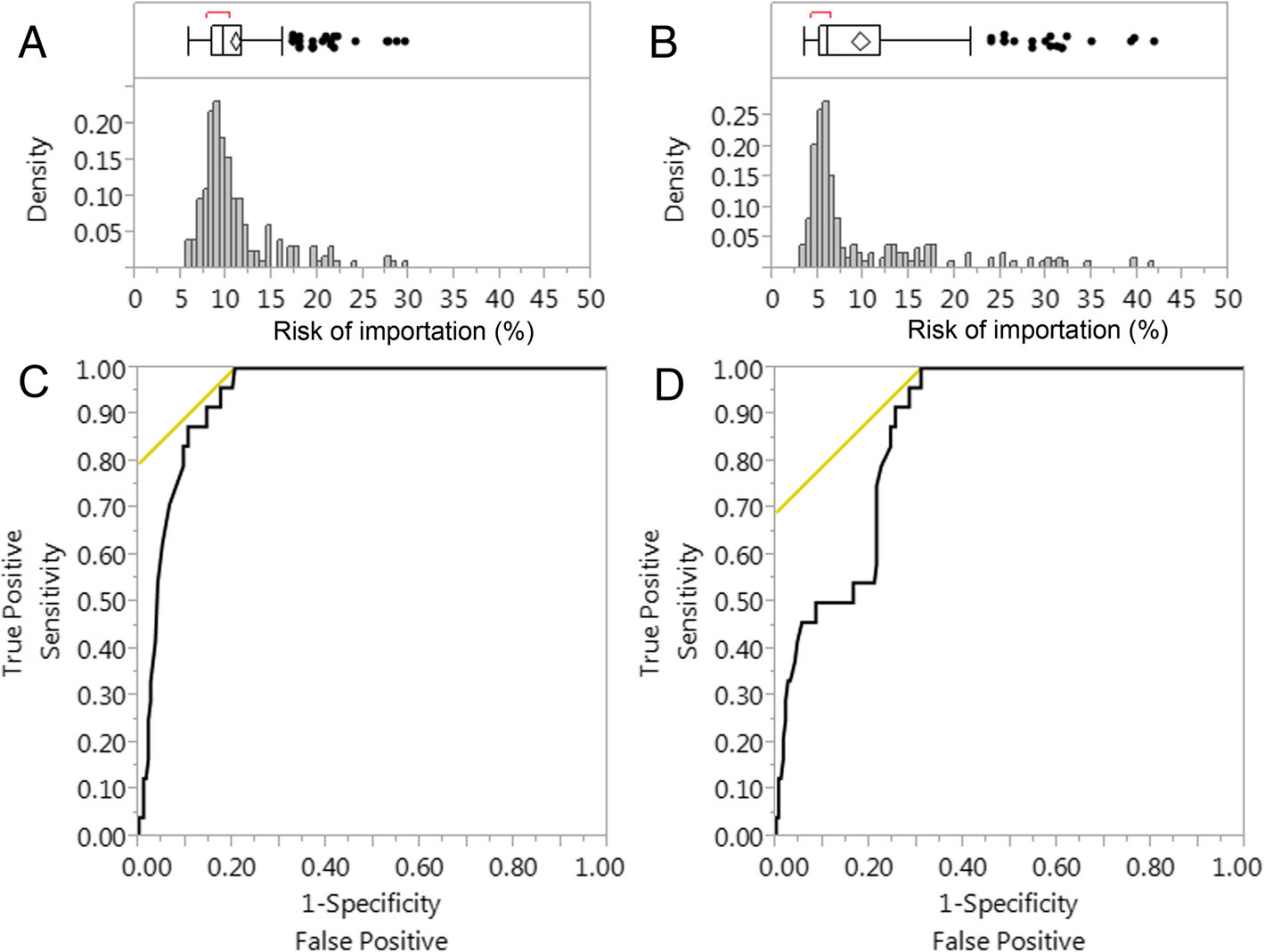
Predicted risk of experiencing a case importation of Middle East respiratory syndrome (MERS). **a** and **b** Distribution of estimated risks of importation by country based on **a** the risk model that used effective distance only and **b** model that used the effective distance as well as religion and incidence data of MERS in the Kingdom of Saudi Arabia. Optimal threshold probability was 11.2 % for panel A and 6.9 % for panel **b**. **c** and **d** Receiver operator characteristic curves of predicted risk of importing MERS cases. Panel **c** shows the evaluation results of the risk model that used effective distance only, while **d** shows those of the model that used the effective distance as well as religion and incidence data of MERS in the Kingdom of Saudi Arabia

Figure 2 shows the predicted risks of MERS importation using two best models, i.e., the simplest model with the largest AUC value (model 1) and the most detailed model with good fit as informed by AIC (model 4). The distribution of predicted risk was skewed to the right, facilitated the visual identification of countries at high risk of experiencing MERS importation in the right tail (Fig. 2a and b). For models 1 and 4, optimal threshold probability to detect countries with importation was estimated at 11.2 and 6.9 %, respectively. When comparing the ROC curves of the two models, AUC of model 1 was greater than that of model 4, indicating that the diagnostic performance of model 4 was inferior at several cut-off values.

**Fig. 3:**
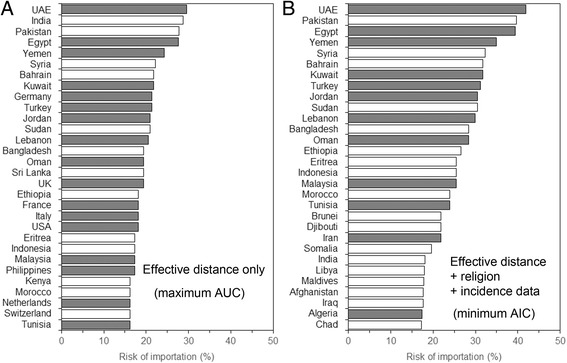
Countries at high risk of case importations of Middle East respiratory syndrome (MERS). List of the 30 countries with the estimated highest importation risks by 3 September 2015. The panel **a** shows the prediction that used the effective distance only with the best predictive value as assessed by the area under the curve (AUC; model 1). The panel **b** shows the prediction using a model that used the effective distance as well as religion and incidence data of MERS in the Kingdom of Saudi Arabia (model 4). The model 4 yielded a smaller value of Akaike Information Criterion (AIC) as compared with the model without incorporating religion information, and was regarded as a model with good fit. Bars filled with black are used to denote those countries that have already experienced at least one MERS importation by 26 June 2015

Figure 3 lists the 30 countries at the highest risk of importing MERS cases by 3 September 2015 (i.e., 3 years since the first detection) using the abovementioned two selected models. Among top-listed 30 countries at highest risk, 17 (56.7 %) have already imported at least one MERS case in model 1 and 12 (40.0 %) have experienced importation in model 4. The difference between two models is understood by comparing countries in panels A and B; panel B includes many Muslim majority countries that have not imported MERS cases. While inclusion of the religion has improved the overall goodness of fit (Table 2), AUC values and Fig. 2 demonstrate that the inclusion did not improve the diagnostic performance of the risk model.

**Fig. 4:**
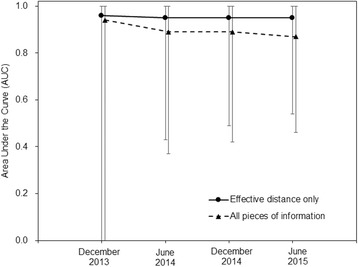
Real-time assessment of the predictive performance of MERS importation. Area Under the Curve (AUC) is compared as a function of time of prediction. Two different models (i.e., effective distance only and the model with all pieces of information) were used, and prediction was performed every six months. Whiskers extend to lower and upper 95 % confidence intervals

Figure 4 shows the results of real-time predictions. There has been no particular time-dependent change in the predictive performance as measured by AUC, but the uncertainty bound (95 % confidence intervals) of AUC has been reduced as a function of calendar time.

## Discussion

The present study devised a novel prediction model to quantify the risk of experiencing a MERS case importation for countries around the world by using data on the arrival time of MERS cases, the global airline transportation network, religion, and the incidence data in Saudi Arabia. We have shown that the proposed model can calculate the country-specific probability of importing MERS cases, using the effective distance with or without accounting for information on religion to classify Muslim majority countries and MERS incidence data. The model is fairly simple compared with more sophisticated simulation approaches [14, 17, 18]. However, all of the analyzed models yielded the sensitivity value of 100 % using cut-off value informed by the Youden index. The simplest model (model 1) with effective distance alone exhibited good predictive performance. The data of Muslim majority countries improved goodness-of-fit and the corresponding countries appeared to have been significantly at an elevated risk of importation, but the predictive performance of models 2 and 4 was lowered compared with models 1 and 3, perhaps elevating the risk of several Muslim majority countries that might actually be very distant from Saudi Arabia. Interestingly, incorporating MERS incidence data into models did not improve their diagnostic performance, although the incidence was added to some of our models to increase the precision of predicted probability of importation.

An important message of the present study is that it is feasible to generate estimates of the risk of importation of MERS using a simplistic and tractable approach using the so-called effective distance as part of the hazard function. As the International Health Regulations (IHR) have never recommended the application of any travel or trade restrictions and screening at points of entry [32–34], countries at risk have had to confront the uncertainty associated with the risk of experiencing a case importation, and we believe that the devised model could facilitate prompt risk assessment practice across countries without the need for complex computational approaches. Especially, since *R*_0_ for MERS is below 1 [33], which is unable to generate large-scale epidemics in the community, with the majority of reported cases likely arising from exposure with an animal reservoir (i.e. dromedary camels) in Middle East countries, our relatively simple approach may act as a good alternative of models that rest on rich assumptions of the transmission dynamics that require more computational resources. The effective distance fully rests on the airline transportation network data and can offer a simplistic approach to this task of prediction. As a consequence, the model 1 correctly included 17 countries (56.6 %) within the top 30 countries at highest risk of importations and that accounts for 70.8 % of the total 24 countries that have already documented MERS case importations. Our model showed fairly good predictability despite its simplicity. The only weakness that has to be noted was the moderate value of specificity, i.e., the proportion true negatives among the total of condition negatives. Identifying one or more predictor variables that could help further discriminate between true positives and false positives could be the subject of future work. As was shown in the relative hazard estimate of Muslim majority countries, one can also examine if a specific group of countries is at significantly high risk of importation compared with other countries.

Using the proposed model, public health authorities across countries could determine where their own country stands in terms of risk relative to other countries. While the present study used the date of analysis (26 June 2015) and 3 years since emergence (3 September 2015) as the dates of prediction, varying the time interval of prediction can be easily attained. Considering that only 24 among 225 countries (10.7 %) have experienced at least one MERS importation, the risk estimate should be updated as further countries may report MERS importations in the future. As the absolute risk estimates are variable by model parameters (as seen in Fig. 2), the risk assessment practice may focus on relative risks and model performance. Risk estimates for individual countries are available upon request.

Due to the simplicity of our approach, several limitations should be explicitly mentioned. First, the probabilistic model essentially assumes random sampling of infected travelers from the population of Saudi Arabia, regardless of nationality and camel contact behavior. While the heterogeneous factors at individual level are highly influential in determining the risk of importation, all mathematical models have so far not attempted to characterize differences between traveler and non-traveler populations. Second, our airline transportation data was based on the country-to-country number of flights and not the itinerary of individual travelers. Therefore, the precision of the effective distance value may also be limited. Nevertheless, the present study gave more weight on the importance of devising simple yet tractable models with the use of publicly available data. Third, to illustrate our approach, we always used Saudi Arabia as the country of origin, but it is worth mentioning that several importation events were associated with surrounding Middle East countries including Qatar, United Arab Emirates, Jordan, Bahrain and Oman. Fourth, the model was country-specific, while the risk of infection is highly heterogeneous within each country [35–37]. Further refinements should be considered in the future.

Despite the number of limitations noted above, it should be stressed out that the proposed model is reproducible by a number of countries, properly detecting all countries that have already imported MERS. We have demonstrated that it is feasible to develop the risk model that predicts specific countries to experience importation with high sensitivity, and the task was achieved by employing an attractive metric, the effective distance, embedded on the hazard model [29]. We are committed to update the model, incorporating additional useful predictors and helping countries across the world to improve on their risk assessment toolkit [38–42].

## Conclusions

We devised a novel statistical model that predicts the risk of importation of MERS case in each country. We collected the arrival time of MERS to each country, dealing with it as a dependent variable to predict. Openly accessible data including the airline transportation network data are used to parameterize a hazard-based risk prediction model. The hazard was assumed as an inverse function of the effective distance calculated from the airline transportation data, and religion and the incidence data of MERS in Saudi Arabia were supplemented to improve the model prediction.

Among the examined models, a model that uses the effective distance only was shown to yield the best predictive performance. Model fit was improved by including country-specific religion (i.e. Muslim majority country), but predictive performance measured by AUC was not improved by accounting for the religion. Our relatively simple statistical model based on the effective distance was found to help predicting the risk of importing MERS at the country level.

## Abbreviations

AIC, Akaike Information Criterion; AUC, Area under the curve; CI, Confidence interval; ECDC, European Centers for Disease Prevention and Control; MERS, Middle East respiratory syndrome; MERS-CoV, Middle East respiratory syndrome associated coronavirus; SARS, Severe acute respiratory syndrome; WHO, World Health Organization

## References

[1] Zumla A, Hui DS, Perlman S. Middle East respiratory syndrome. Lancet. 2015. doi: 10.1016/S0140-6736(15)60454-8. 10.1016/S0140-6736(15)60454-8 PMC4721578 26049252

[2] Assiri A, McGeer A, Perl TM, Price CS, Al Rabeeah AA, Cummings DA, Alabdullatif ZN, Assad M, Almulhim A, Makhdoom H, Madani H, Alhakeem R, Al-Tawfiq JA, Cotten M, Watson SJ, Kellam P, Zumla AI, Memish ZA. Hospital outbreak of Middle East respiratory syndrome coronavirus. N Engl J Med. 2013;369(5):407–16. 10.1056/NEJMoa1306742 PMC4029105 23782161

[3] Chowell G, Blumberg S, Simonsen L, Miller MA, Viboud C (2014). Synthesizing data and models for the spread of MERS-CoV, 2013: key role of index cases and hospital transmission. Epidemics.

[4] Cauchemez S, Fraser C, Van Kerkhove MD, Donnelly CA, Riley S, Rambaut A, Enouf V, van der Werf S, Ferguson NM (2014). Middle East respiratory syndrome coronavirus: quantification of the extent of the epidemic, surveillance biases, and transmissibility. Lancet Infect Dis.

[5] Breban R, Riou J, Fontanet A (2013). Interhuman transmissibility of Middle East respiratory syndrome coronavirus: estimation of pandemic risk. Lancet.

[6] Meyer B, Müller MA, Corman VM, Reusken CB, Ritz D, Godeke GJ, Lattwein E, Kallies S, Siemens A, van Beek J, Drexler JF, Muth D, Bosch BJ, Wernery U, Koopmans MP, Wernery R, Drosten C (2014). Antibodies against MERS coronavirus in dromedary camels, United Arab Emirates, 2003 and 2013. Emerg Infect Dis.

[7] Müller MA, Meyer B, Corman VM, Al-Masri M, Turkestani A, Ritz D, Sieberg A, Aldabbagh S, Bosch BJ, Lattwein E, Alhakeem RF, Assiri AM, Albarrak AM, Al-Shangiti AM, Al-Tawfiq JA, Wikramaratna P, Alrabeeah AA, Drosten C, Memish ZA (2015). Presence of Middle East respiratory syndrome coronavirus antibodies in Saudi Arabia: a nationwide, cross-sectional, serological study. Lancet Infect Dis.

[8] Hui DS, Perlman S, Zumla A. Spread of MERS to South Korea and China. Lancet Respir Med 2015. doi: 10.1016/S2213-2600(15)00238-6. 10.1016/S2213-2600(15)00238-6 PMC7128695 26050550

[9] Cowling BJ, Park M, Fang VJ, Wu P, Leung GM, Wu JT. Preliminary epidemiological assessment of MERS-CoV outbreak in South Korea, May to June 2015. Eurosurveillance 2015;20(25). Available online: http://www.eurosurveillance.org/ViewArticle.aspx?ArticleId=21163 10.2807/1560-7917.es2015.20.25.21163 PMC4535930 26132767

[10] World Health Organization (WHO). Middle East respiratory syndrome coronavirus (MERS-CoV) Geneva, Switzerland: WHO; 2015. (Available from: [http://www.who.int/mediacentre/factsheets/mers-cov/en/]) (Last accessed on: 27 June 2015)

[11] Kucharski AJ, Althaus CL. The role of superspreading in Middle East respiratory syndrome coronavirus (MERS-CoV) transmission. Eurosurveillance 2015;20(25). Available online: http://www.eurosurveillance.org/ViewArticle.aspx?ArticleId=21167 10.2807/1560-7917.es2015.20.25.21167 26132768

[12] Colizza V, Barrat A, Barthélemy M, Vespignani A (2006). The role of the airline transportation network in the prediction and predictability of global epidemics. Proc Natl Acad Sci U S A.

[13] Balcan D, Hu H, Goncalves B, Bajardi P, Poletto C, Ramasco JJ, Paolotti D, Perra N, Tizzoni M, Van den Broeck W, Colizza V, Vespignani A (2009). Seasonal transmission potential and activity peaks of the new influenza A(H1N1): a Monte Carlo likelihood analysis based on human mobility. BMC Med.

[14] Gomes MFC, Pastore y Piontti A, Rossi L, Chao D, Longini I, Halloran ME, Vespignani A. Assessing the International Spreading Risk Associated with the 2014 West African Ebola Outbreak PLOS Currents Outbreaks. 2014; Sep 2. Edition 1. doi: 10.1371/currents.outbreaks.cd818f63d40e24aef769dda7df9e0da5. 10.1371/currents.outbreaks.cd818f63d40e24aef769dda7df9e0da5 PMC4169359 25642360

[15] Bogoch II, Creatore MI, Cetron MS, Brownstein JS, Pesik N, Miniota J, Tam T, Hu W, Nicolucci A, Ahmed S, Yoon JW, Berry I, Hay SI, Anema A, Tatem AJ, MacFadden D, German M, Khan K (2015). Assessment of the potential for international dissemination of Ebola virus via commercial air travel during the 2014 west African outbreak. Lancet.

[16] Poletto C, Gomes MF, Pastore y Piontti A, Rossi L, Bioglio L, Chao DL, Longini IM, Halloran ME, Colizza V, Vespignani A. Assessing the impact of travel restrictions on international spread of the 2014 West African Ebola epidemic. Euro Surveill. 2014;19(42). 10.2807/1560-7917.es2014.19.42.20936 PMC4415609 25358040

[17] Khan K, Sears J, Hu VW, Brownstein JS, Hay S, Kossowsky D, Eckhardt R, Chim T, Berry I, Bogoch I, Cetron M. Potential for the international spread of middle East respiratory syndrome in association with mass gatherings in saudi arabia. PLoS Curr 2013; 5. doi: 10.1371/currents.outbreaks.a7b70897ac2fa4f79b59f90d24c860b8. 10.1371/currents.outbreaks.a7b70897ac2fa4f79b59f90d24c860b8 PMC3714242 23884087

[18] Lessler J, Rodriguez-Barraquer I, Cummings DA, Garske T, Van Kerkhove M, Mills H, Truelove S, Hakeem R, Albarrak A, Ferguson NM; MERS-CoV Scenario Modeling Working Group; MERS-CoV Scenario Modeling Working Group. Estimating Potential Incidence of MERS-CoV Associated with Hajj Pilgrims to Saudi Arabia, 2014. PLoS Curr. 2014;6. doi: 10.1371/currents.outbreaks.c5c9c9abd636164a9b6fd4dbda974369. 10.1371/currents.outbreaks.c5c9c9abd636164a9b6fd4dbda974369 PMC4323406 25685624

[19] European Centres for Disease Prevention and Control (ECDC) (2015). Severe respiratory diseases associated with Middle East respiratory syndrome coronavirus (MERS-CoV)I. Fifteenth update – 8 March 2015.

[20] World Health Organization (WHO) Disease Outbreak News (DONs) Geneva, Switzerland: WHO; 2015. (Available from: [http://www.who.int/csr/don/en/index.html]) (last accessed on: 30 June 2015)

[21] Reuss A, Litterst A, Drosten C, Seilmaier M, Böhmer M, Graf P, Gold H, Wendtner CM, Zanuzdana A, Schaade L, Haas W, Buchholz U (2014). Contact investigation for imported case of Middle East respiratory syndrome, Germany. Emerg Infect Dis.

[22] Yavarian J, Rezaei F, Shadab A, Soroush M, Gooya MM, Azad TM (2015). Cluster of Middle East respiratory syndrome coronavirus infections in Iran, 2014. Emerg Infect Dis.

[23] Buchholz U, Müller MA, Nitsche A, Sanewski A, Wevering N, Bauer-Balci T, Bonin F, Drosten C, Schweiger B, Wolff T, Muth D, Meyer B, Buda S, Krause G, Schaade L, Haas W. Contact investigation of a case of human novel coronavirus infection treated in a German hospital, October-November 2012. Euro Surveill. 2013;18(8). 23449231

[24] World Health Organization (WHO) Middle East respiratory syndrome coronavirus (MERS-CoV). Geneva, Switzerland: WHO; 2015. (Available from: [http://www.who.int/emergencies/mers-cov/en/]) (last accessed on: 30 June 2015)

[25] Visualizing.org. Global Flights Network. USA: General Electric Company and Seed Media Group (Available from: [http://www.visualizing.org/datasets/global-flights-network]) (Last accessed on: 28 June 2015)

[26] Contentshare. Openflight database. Singapore: Contentshare (Available from: [http://openflights.org]) (Last accessed on: 27 June 2015)

[27] Hackett C, Grim B, Stonawski M, Skirbekk V, Potančoková M, Abel G (2012). The Global Religious Landscape.

[28] Pebody RG, Chand MA, Thomas HL, Green HK, Boddington NL, Carvalho C, Brown CS, Anderson SR, Rooney C, Crawley-Boevey E, Irwin DJ, Aarons E, Tong C, Newsholme W, Price N, Langrish C, Tucker D, Zhao H, Phin N, Crofts J, Bermingham A, Gilgunn-Jones E, Brown KE, Evans B, Catchpole M, Watson JM (2012). The United Kingdom public health response to an imported laboratory confirmed case of a novel coronavirus in September 2012. Euro Surveill.

[29] Brockmann D, Helbing D (2013). The hidden geometry of complex, network-driven contagion phenomena. Science.

[30] Akaike H (1981). Likelihood of a model and information criteria. J Econ.

[31] Greiner M, Pfeiffer D, Smith RD (2000). Principles and practical application of the receiver-operating characteristic analysis for diagnostic tests. Prev Vet Med.

[32] World Health Organization (WHO). IHR Emergency Committee concerning Middle East respiratory syndrome coronavirus. Geneva, Switzerland: WHO; 2015. (Available from: [http://www.who.int/ihr/ihr_ec_2013/en/]) (last accessed on: 30 June 2015)

[33] Nishiura H, Miyamatsu Y, Chowell G, Saitoh M. Assessing the risk of observing multiple generations of Middle East respiratory syndrome (MERS) cases given an imported case. Euro Surveill 2015;20(27). 10.2807/1560-7917.es2015.20.27.21181 26212063

[34] Nishiura H, Miyamatsu Y, Mizumoto K (2016). Objective Determination of End of MERS Outbreak, South Korea, 2015. Emerg Infect Dis.

[35] Merler S, Ajelli M, Fumanelli L, Gomes MF, Piontti AP, Rossi L, Chao DL, Longini IM, Halloran ME, Vespignani A (2015). Spatiotemporal spread of the 2014 outbreak of Ebola virus disease in Liberia and the effectiveness of non-pharmaceutical interventions: a computational modelling analysis. Lancet Infect Dis.

[36] Wesolowski A, Buckee CO, Bengtsson L, Wetter E, Lu X, Tatem AJ. Commentary: containing the ebola outbreak - the potential and challenge of mobile network data. PLoS Curr. 2014 Sep 29;6. doi: 10.1371/currents.outbreaks.0177e7fcf52217b8b634376e2f3efc5e. 10.1371/currents.outbreaks.0177e7fcf52217b8b634376e2f3efc5e PMC4205120 25642369

[37] Nah K, Mizumoto K, Miyamatsu Y, Yasuda Y, Kinoshita R, Nishiura H (2016). Estimating risks of importation and local transmission of Zika virus infection. Peer J.

[38] Nishiura H, Endo A, Saitoh M, Kinoshita R, Ueno R, Nakaoka S, Miyamatsu Y, Dong Y, Chowell G, Mizumoto K (2016). Identifying determinants of heterogeneous transmission dynamics of the Middle East respiratory syndrome (MERS) outbreak in the Republic of Korea, 2015: a retrospective epidemiological analysis. BMJ Open.

[39] Mizumoto K, Saitoh M, Chowell G, Miyamatsu Y, Nishiura H (2015). Estimating the risk of Middle East respiratory syndrome (MERS) death during the course of the outbreak in the Republic of Korea, 2015. Int J Infect Dis.

[40] Mizumoto K, Endo A, Chowell G, Miyamatsu Y, Saitoh M, Nishiura H (2015). Real-time characterization of risks of death associated with the Middle East respiratory syndrome (MERS) in the Republic of Korea, 2015. BMC Med.

[41] Nishiura H, Kinoshita R, Mizumoto K, Yasuda Y, Nah K (2016). Transmission potential of Zika virus infection in the South Pacific. Int J Infect Dis.

[42] Nishiura H, Mizumoto K, Rock KS, Yasuda Y, Kinoshita R, Miyamatsu Y. A theoretical estimate of the risk of microcephaly during pregnancy with Zika virus infection. Epidemics. 2016;15:66-70. doi:10.1016/j.epidem.2016.03.001. 10.1016/j.epidem.2016.03.001 27288540

